# ChEBI in 2016: Improved services and an expanding collection of metabolites

**DOI:** 10.1093/nar/gkv1031

**Published:** 2015-10-13

**Authors:** Janna Hastings, Gareth Owen, Adriano Dekker, Marcus Ennis, Namrata Kale, Venkatesh Muthukrishnan, Steve Turner, Neil Swainston, Pedro Mendes, Christoph Steinbeck

**Affiliations:** 1 Cheminformatics and Metabolism, European Molecular Biology Laboratory—European Bioinformatics Institute (EMBL-EBI), Hinxton, UK; 2 Manchester Centre for Integrative Systems Biology, University of Manchester, UK

## Abstract

ChEBI is a database and ontology containing information about chemical entities of biological interest. It currently includes over 46 000 entries, each of which is classified within the ontology and assigned multiple annotations including (where relevant) a chemical structure, database cross-references, synonyms and literature citations. All content is freely available and can be accessed online at http://www.ebi.ac.uk/chebi. In this update paper, we describe recent improvements and additions to the ChEBI offering. We have substantially extended our collection of endogenous metabolites for several organisms including human, mouse, *Escherichia coli* and yeast. Our front-end has also been reworked and updated, improving the user experience, removing our dependency on Java applets in favour of embedded JavaScript components and moving from a monthly release update to a ‘live’ website. Programmatic access has been improved by the introduction of a library, libChEBI, in Java, Python and Matlab. Furthermore, we have added two new tools, namely an analysis tool, BiNChE, and a query tool for the ontology, OntoQuery.

## INTRODUCTION

ChEBI is a database and ontology of chemical entities of biological interest containing a wide range of manually curated data items (1–3). Each entry in the database is classified within the ontology. There are two main sub-ontologies, namely a chemical entity ontology in which chemical entities are classified based on shared structural features, and a role ontology in which entities are classified based on their activities in biological or chemical systems or their use in applications. The primary ontology relationships are the ‘is a’ relationship for classification, the ‘has role’ relationship which links chemical entities to their roles and ‘has part’ which links composite entities, e.g. salts to their component parts. Several additional ontology relationships are used to represent, e.g. tautomers, enantiomers and other chemistry-specific interrelationships. Each entry in the database is assigned several metadata annotations, including where relevant a representation of the chemical structure, cross-references to other databases, multiple synonyms and alternative names in other languages, species in which a particular entity has been found and literature citations. ChEBI includes additional text from Wikipedia where possible (in turn, Wikipedia contains links back to ChEBI from their chemistry articles) and the monthly ‘Entity of the month’ article (http://www.ebi.ac.uk/chebi/entityMonthForward.do) is a popular feature in which a particular entry in the database is highlighted in the context of a recent scientific discovery or appearance in the popular press.

As of the most recent release (September 2015), ChEBI includes 46 477 fully-curated entries, of which 7360 were submitted directly by users via our online submission tool. The database further includes a backlog of 9340 entries which have been loaded from relevant external collections, as described further below. Additional details about the current content are available from our statistics page available at http://www.ebi.ac.uk/chebi/statisticsForward.do. All content in ChEBI is freely available for any use and can be accessed online at http://www.ebi.ac.uk/chebi.

ChEBI is widely used for many different purposes including as a source of stable unique identifiers for chemicals in annotations in a wide range of bioinformatics databases, including as of recently UniProt for references to chemicals as cofactors, (4) and systems biology models (5,6). It is also used as a knowledge base for text and data mining purposes (7,8), and as the chemistry component of several ontologies including the popular Gene Ontology (9). Furthermore, ChEBI is used in the context of the Semantic Web, for example the recent representation of the PubChem database content as RDF used ChEBI classes where possible to provide the rdf:type classification for the PubChem chemicals in their RDF representation (10).

In this update paper we describe recent improvements and additions to the ChEBI offering over and above those reported on previously (1–3). In the next section, we describe how we have substantially extended our collection of endogenous metabolites for several organisms. The following section reports on updates to our user interface, in which we have improved the user experience, removed our dependency on Java applets in favour of embedded JavaScript components and moved from monthly updates to a ‘live’ content stream. Finally, we describe two new tools that have been added to our online software suite, namely an analysis tool, BiNChE, and an ontology query tool, OntoQuery.

## METABOLISM COLLECTION

Since our previous report (3), a concerted effort in curation has resulted in a >50% increase in the number of fully curated entries in ChEBI. Curation has focused on natural products (‘metabolites’), leading to a substantial extension to ChEBI's collection of endogenous metabolites for several organisms, including human, mouse, *Escherichia coli* and yeast. In total, ChEBI now (as of September 2015) contains 14 489 metabolites across 2114 distinct species.

**Figure 1.**
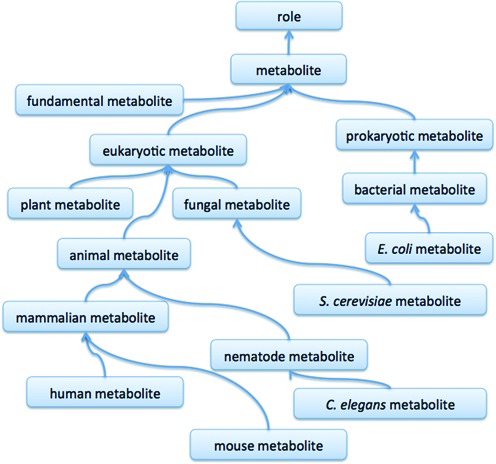
The organisation of a subset of the metabolite ontology classification in ChEBI, illustrating the hierarchical (‘is a’) classification of different types of metabolite, including those which have been significant in our curation efforts such as *E.coli* and human metabolite.

In order to better represent these metabolites within ChEBI, we have refactored and extended the ontology classification of metabolites. ‘Metabolite’ is a class in the role ontology; beneath ‘metabolite’ we have classes such as ‘eukaryotic metabolite’ and ‘prokaryotic metabolite’ as well as ‘fundamental metabolite’ which are those that are common to all living species. The actual metabolites are classified in the chemical entity ontology based on their structural features and then linked to the relevant metabolite classes via the ‘has role’ relationship. An overview of a subset of the metabolite classification in ChEBI is illustrated in Figure 1.

For metabolites included in ChEBI, the reference layer of our partner database MetaboLights (11) includes in some cases additional metabolically relevant information for the chemical, such as reference NMR and MS spectra where available, as well as links to experiments in the MetaboLights repository in which that chemical has been measured in a sample.

As the number of metabolites in ChEBI has increased, so sections of the ChEBI ontology have been extended, revised and reorganised to improve searching and reflect current nomenclature recommendations. Thus the classification over 2200 flavonoids has been revisited and updated to reflect the latest IUPAC proposals for their nomenclature (12), with initial subdivisions to cover aurones, chalcones, coumestans, dihydrochalcones, flavonoids, flavonoid oligomers, flavonolignans, homoflavonoids, isoflavonoids, neoflavonoids, pterocarpans and rotenoids. In a similar manner, in response to user requests to help distinguish between the (relatively few) amino acids that are particularly important in life processes from the large number of other amino acids that occur much less frequently, the ontology branch containing amino acids, with more than 800 dependent entries has now been divided into proteinogenic amino acids (i.e. the 20 alpha-amino acids that are encoded by the nuclear genes of eukaryotes together with selenocysteine, pyrrolysine and N-formylmethionine), and non-proteinogenic amino acids, the latter being further divided according to the position of the amino group (α-, β-, γ-, etc.).

Additional cross-references of relevance to the representation of metabolism have been added, including to the Human Metabolome database, HMDB (13), the Golm metabolome database (14), MassBank (15), KNApSAcK (16), UM-BBD (17), SMID (18) and the Yeast Metabolome database, YMDB (19). In many of these cases, to speed up the curation of novel content into ChEBI, additional small molecule metabolite information contained in the databases and not yet present in ChEBI was loaded into the ChEBI pre-curation collection using a pipeline for the automated loading and classification of high-quality externally curated content.

## SOFTWARE UPDATES

The ChEBI public web application has been reworked and updated, with an emphasis on page layout, responsiveness, menu structure and an overall improvement in the interactive user experience. An important aspect of this renovation was to replace all the Java applets that were in use with appropriate JavaScript components and libraries. Java applets have been a perennial source of user frustration in the past, as they tended to operate differently on different user platforms and to be bulky to download, hindering usage from slow connections. There were also security concerns in the use of Java in web browsers, and from time to time browser security settings would block certain of the applets from executing.

**Figure 2.**
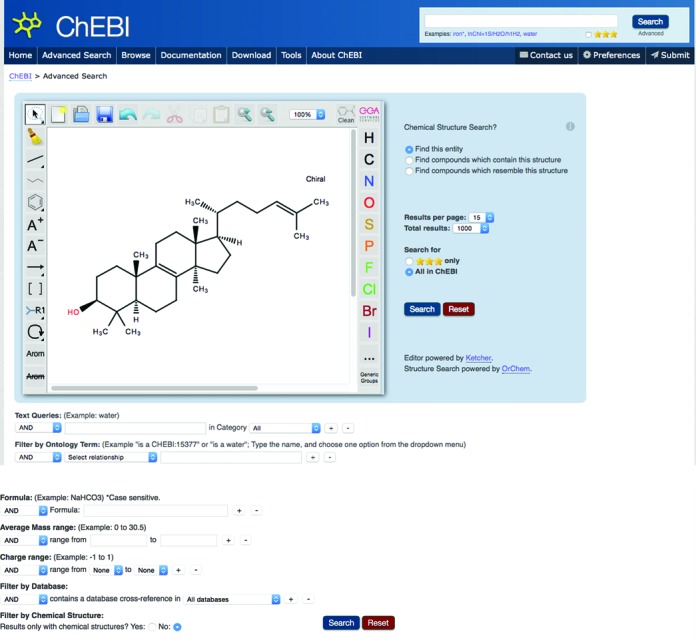
A screenshot of the new ChEBI advanced search page interface, showing the JavaScript structure sketcher Ketcher, and the new menu options along the top bar, which also includes a search box.

To address all of these concerns, we have harnessed 100% JavaScript alternatives for all of the previously applet-tied functionality, primarily around the chemical structure editing (for searching) and visualisation. The JavaScript chemical structure editor we are using is Ketcher (20), as illustrated in Figure 2, and for 3D structure viewing we are using JSmol (http://sourceforge.net/projects/jsmol/).

We have also improved our submitter tool, similarly replacing applets with JavaScript components, and further adding an option for bulk submissions by SDfile, wherein any associated data properties such as cross-references, synonyms etc. can flexibly be mapped to ChEBI data columns. Each entry in a bulk submission must have a unique name, an ontology classification and a unique chemical structure. A definition is highly recommended, as are associated metadata such as synonyms and cross-references. We are currently working on extending the bulk submission facility with the possibility of automatically classifying new entries within the ontology, which would render the provided ontology classification non-mandatory.

To further enhance user experience, particularly that of submitters and others who use ChEBI IDs in data or text annotations, we have moved from the public website content being updated as a part of our monthly release cycle to a ‘live’ website. This means that as soon as an entry is submitted via the submission tool, a permanent ChEBI ID has been allocated and the entry is also visible in the public ChEBI web interface. Similarly, curator-added content and changes are visible as soon as they are saved to the underlying database. This obviates the need for a release cycle imposed delay in making use of newly allocated IDs, and furthermore allows content corrections to be immediately visible. The search index is updated overnight, while the download files, in various formats, along with the Entity of the Month article, are still updated monthly, with the release taking place on the first day of every month.

To ease programmatic access to ChEBI content and to seamlessly enable the use of ChEBI content in software applications, for example in systems biology and metabolic modelling contexts, we have created a library for programmatic access, libChEBI. The library is fully open source and is available in Java, Python and Matlab. Source code is available from https://github.com/libChEBI.

## ADDITIONAL TOOLS

### BiNChE

One of the most important applications for ontologies such as the Gene Ontology is in the category-based statistical analysis of differential enrichment in large-scale data sets arising from modern high-throughput measurements. Although many such tools exist for the Gene Ontology, there were relatively few dealing with the analysis of chemicals using the ChEBI ontology. We have created a web-based enrichment analysis tool, BiNChE, available from http://www.ebi.ac.uk/chebi/tools/binche/ (21), which is also available as a software library. The tool offers plain or weighted analysis options against the ChEBI role, structure or combined ontology.

**Figure 3.**
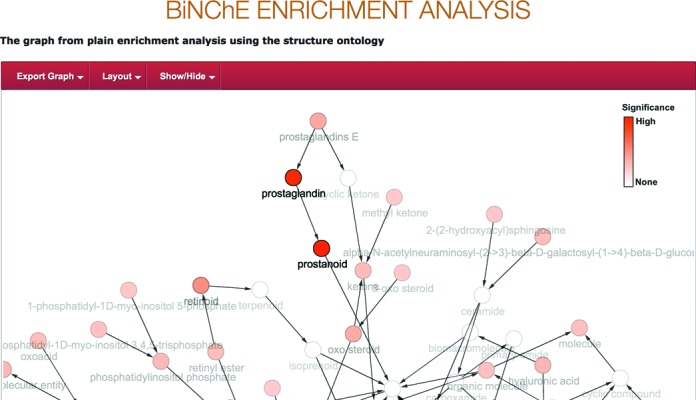
A screenshot of the result of plain enrichment analysis in BiNChE with the ChEBI structure ontology. The interface is draggable and zoomable. The intensity of the colour indicates the significance of the enrichment at each node.

The input to the tool is a set of ChEBI IDs, together in the weighted case with a set of weights which are values between 0 and 1. The output is displayed as a graph but may also be downloaded in several formats including as a table. BiNChE is intended to support the analysis of results in metabolomics experiments, for example in making sense of results in which different sets of metabolites are observed under different experimental conditions. Figure 3 shows a screenshot of the BiNChE interface for a given sample set of input ChEBI IDs against the ChEBI structure ontology.

### OntoQuery

For the easy formulation and execution of complex logical queries against the ontology, we have provided a web-based tool, OntoQuery, available from http://www.ebi.ac.uk/chebi/tools/ontoquery/ (22) which allows Description Logic queries in the easy to use Manchester syntax (23) to be executed against the pre-loaded and pre-reasoned ChEBI ontology.

**Figure 4.**
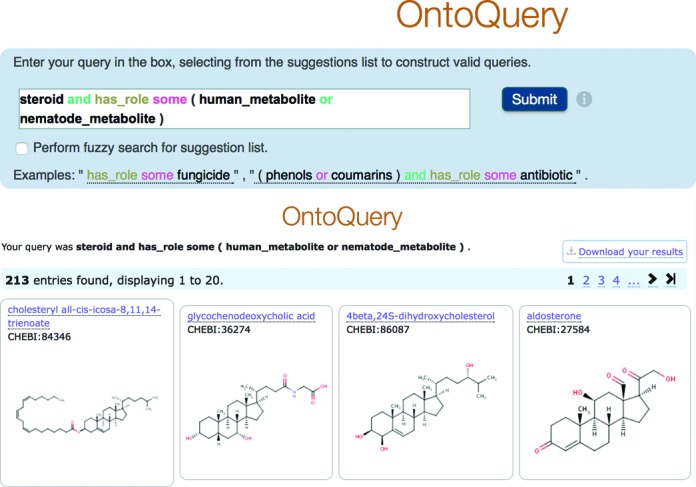
A screenshot of a sample query and result page from the OntoQuery tool.

OntoQuery offers syntax suggestions and corrections as you type, and supports queries over any logical combination (using ‘and’, ‘or’) of classes or relationships in the ontology. The OntoQuery interface is illustrated in Figure 4, showing a sample query and its results.

## CONCLUSION

As indicated by widespread adoption, ChEBI has become an essential bioinformatics chemistry database, enabling multiple diverse applications for different types of user in various scientific contexts. In this update, we report on several new features and enhancements which have been added to ChEBI in the recent years since our last publication.
